# Progressive Fatal Myelopathy Secondary to Isolated Spinal Cord Vasculitis

**DOI:** 10.3389/fneur.2017.00705

**Published:** 2017-12-19

**Authors:** Arie Fisher, Habib Rahman, Michael Farrell, Michael Hennessy

**Affiliations:** ^1^Department of Neurology, University Hospital Galway, Galway, Ireland; ^2^Department of Histopathology, Beaumont Hospital, Dublin, Ireland

**Keywords:** primary angiitis of the central nervous system, CNS vasculitis, spinal cord vasculitis, spinal cord angiitis, CNS angiitis, myelopathy, spinal arteritis, spinal inflammation

## Abstract

A 56-year-old woman with various neurological signs which developed over a 1-year period was admitted for evaluation. MRI showed a markedly abnormal signal in the cervical spine. Despite treatment with IV steroids, she developed a progressive myelopathy, became quadriplegic, and required intubation. Immunomodulatory treatment was ineffective. The patient died 24 days after admission. Histopathological investigation revealed spinal cord necrosis with a lymphocyte predominant meningovascular inflammation involving arteries and veins along with evidence of prior occlusive disease of the anterior spinal artery. The changes were confined to the spinal cord. The present case represents an unusual cause of myelitis for which early and aggressive immunomodulatory treatment may have influenced outcomes.

## Introduction

Brain and spinal cord may be involved by an array of systemic vasculitides which include systemic lupus erythematosus, Wegner’s granulomatosis, polyarteritis nodosa, Churg–Strauss angiitis, Behçets syndrome, and Sjögren’s syndrome (1). By contrast, vasculitis confined to the brain and spinal cord is extremely rare but is best described in the entity termed primary angiitis of the central nervous system (PACNS). Diagnosis of PACNS requires (1) the presence of an acquired otherwise unexplained neurological or psychiatric deficit; (2) the presence of either classic angiographic or histopathological features of angiitis within the CNS; and (3) no evidence of systemic vasculitis or any disorder that could cause or mimic the angiographic or pathological features of the disease. The lack of specific and sensitive testing makes diagnosis elusive.

## Background

A 56-year-old woman with a past medical history of fibromyalgia and hypertension presented to an outpatient service with poor balance, ataxic gait, hyperreflexia, and right-sided blurred vision. Head MRI showed non-specific white matter changes, more consistent with microvascular ischemic disease than with demyelination. Whole spine MRI showed a small area of hyperintensity in the spinal cord at T10/T11. Oligoclonal bands were present in both CSF and serum. Treatment with IV methylprednisolone 1,000 mg was given over 5 days for suspected transverse myelitis. Over the course of the next year, balance worsened and limb paresthesia together with urinary incontinence and anorexia developed culminating in a fall which required hospitalization.

### Hospital Course

**Figure 1:**
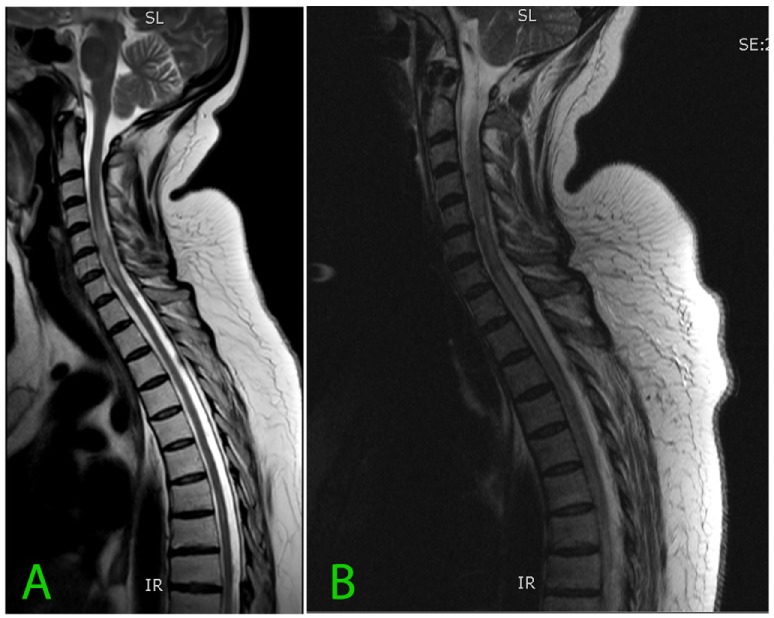
**(A)** Sagittal T2 sequence and **(B)** follow-up MRI a month later demonstrating signal abnormality within the cervical cord. The differential diagnosis of abnormal cord signal depends on the localization. Short segments are typical for multiple sclerosis, trauma, and progressive myelopathy, but long segment abnormalities represent a wider range of mostly uncommon conditions including transverse myelitis, neuromyelitis optica, dural arteriovenous fistula, arterial ischemia, sarcoidosis, vasculitis, West Nile virus, HIV, and Vitamin B deficiency (2).

A head MRI was unchanged and cervical spine MRI revealed a markedly abnormal signal (Figure 1). She was again treated with IV methylprednisolone for a presumed diagnosis of transverse myelitis. Two weeks later, there was sudden onset lower limb paraparesis with loss of pain and temperature sensation in the lower limb, dyspnea, oxygen desaturation, urinary retention, and alternating levels of consciousness developed. Eventually the patient became quadriplegic. IV Dexamethasone 24 mg daily, acyclovir, and antibiotics were commenced, and the patient was intubated and ventilated.

CSF analysis showed glucose 2.3 mmol/L (41.44 mg/dL), protein 1.12 g/L, WBC 1,030 cells/μL, with 56% polymorphonuclear cells without malignant cells. Repeat CSF examination after treatment showed reduced pleocytosis, WBC 12 cells/μL (of which 99% were lymphocytes), protein 0.84 g/L, and glucose 4.5 mmol/L (81 mg/dL) but clinical improvement did not occur.

Repeat MRI (Figure 1) was performed 4 weeks after the previous cervical spine MRI and revealed progression of the abnormal signal, which at that time extended from C2 to T7 with cord edema.

The following investigations were normal or negative: anti-NMO antibodies in serum and CSF, anti nuclear antibodies including antibodies directed against the antigens SM, SSA/Ro, SSB/La, U1RNP, Centromere, Scl-70 and Jol, anticardiolipin, ANCA, anti Ri, Hu, and Yo, and anti-thyroglobulin. PCR for HBV and HCV, HSV1/HSV2, and enterovirus in the CSF were negative. Cryptococcal antigen was not detected in the CSF. CSF culture was sterile and angiotensin-converting enzyme was not present in the CSF.

Deterioration continued despite aggressive treatment with plasmapheresis and cyclophosphamide (1,200 mg given 11 days following admission). 14 days after the extent of the lesion was demonstrated on imaging, it was felt that all possible therapeutic options have been explored and that at that stage the patient’s condition, irrespective of causation, was almost certainly irreversible. The patient’s next of kin was of the view that she would not wish to survive without a meaningful prospect of recovery and that she would wish for a tracheostomy to be avoided. Palliative treatment was initiated and the patient was extubated. Death from neurogenic respiratory failure occurred 24 days after admission.

### Histopathological Examination

**Figure 2:**
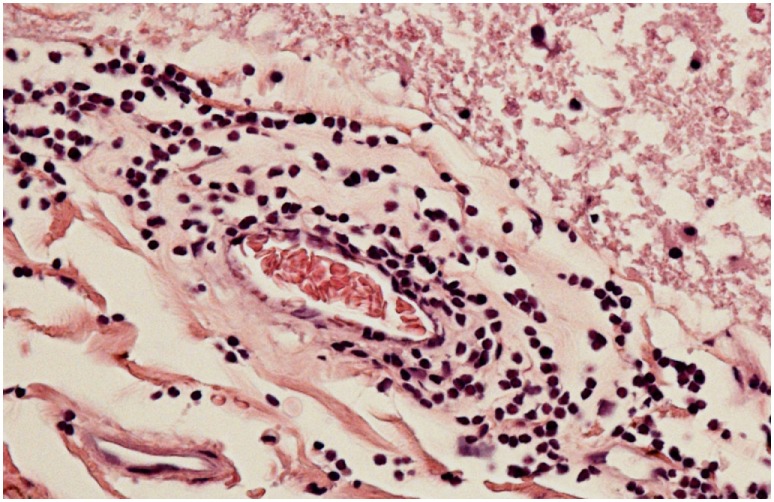
Meningeal vessel showing intense transmural lymphocytic infiltrate with patent lumen.

**Figure 3:**
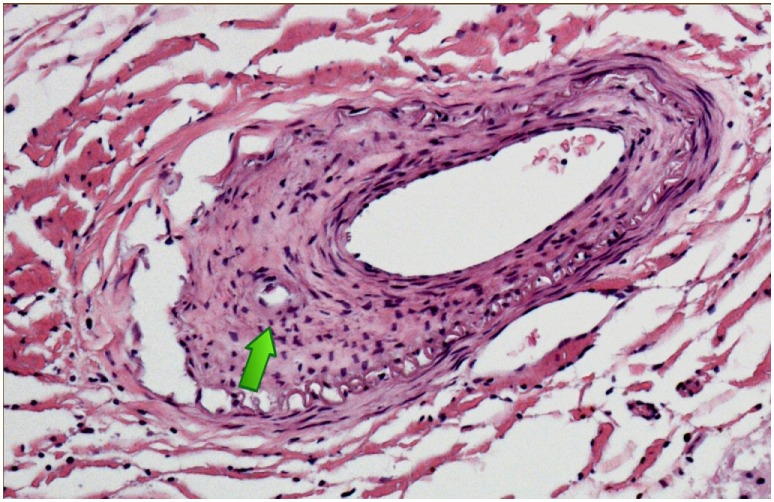
Anterior spinal artery cervical spinal cord showing evidence of prior occlusion in the form of proliferating medial myofibroblasts with recanalization [arrow].

**Figure 4:**
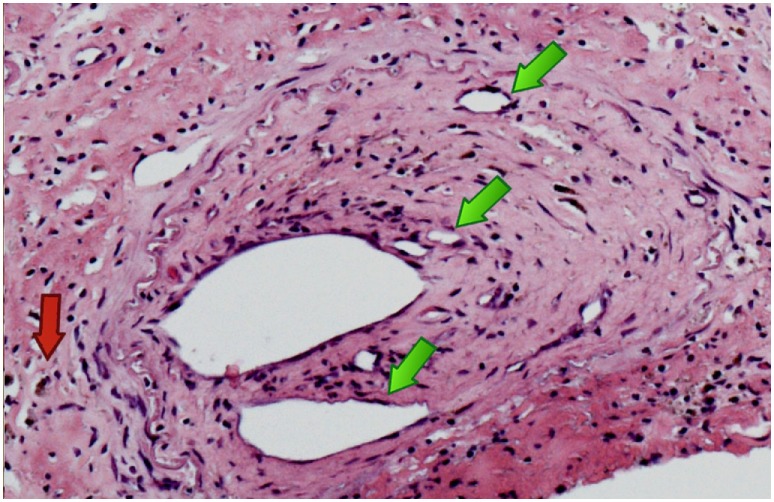
Anterior spinal artery at a different cervical level again showing recanalization with multiple new channels [green arrows] with hemosiderin deposition [red arrow].

Neuropathologic examination showed pan-medullary necrosis with rostral extension to the cervicomedullary junction and caudal extension on either side of the area of necrosis. There was a minor degree of pallor involving the fasciculus gracilis and fasciculus cuneatus in the cervical region but no other abnormalities were observed. In the mid cervical region, however, there was virtually complete pan-medullary necrosis in which virtually all of the spinal cord including gray and white matter showed cavitation with replacement by large macrophages. At the interface between the residual intact spinal cord and the central necrotic area, the macrophages were prominent and distended with lipid. Additionally, there were numerous axonal spheroids present at the interface between the central necrotic area and the adjacent rim of intact non necrotic spinal cord. There was a light-associated meningeal lymphocytic infiltrate. In several areas, lymphocytes were prominent in a perivenous distribution and in one vessel at least the lymphocytes extended across the vessel wall. The lymphocytes were not atypical. Plasma cells were not evident. More caudally, there was clear and unequivocal evidence of a meningovascular inflammatory process dominated by lymphocytes and involving both arteries and veins (Figure 2) but at this level spinal necrosis was not evident. There was also evidence of prior vascular occlusion involving the anterior spinal artery in at least two levels with revascularization (Figures 3 and 4). Thrombosis and inflammation were not identified involving the anterior spinal artery but the artery showed evidence of recanalization in the form of small intramural vascular channels as well as old intramural hemorrhage in the form of hemosiderin deposits. The vascular changes are consistent with the patient’s spinal cord necrosis and the histologic changes point toward an event having occurred 2–5 weeks previously. There was no evidence of vasculitis in tissues outside the spinal cord. There were no significant abnormalities in the patient’s brain.

## Discussion

We report the clinical and pathologic features of isolated primary spinal vasculitis which was not associated with systemic vasculitis or with any known vasculitis immune perturbations.

The differential diagnosis of myelitis is wide and includes inflammatory, infectious, vascular, and neoplastic diseases. It was felt that the subacute course, imaging findings of long segment signal abnormality with cord edema and CSF findings of elevated protein and pleocytosis were most suggestive of inflammatory disease. Neuromyelitis optica was particularly suspected. In this inflammatory demyelinating syndrome, attacks occur in the spinal cord, optic nerves, or in both these sites. The condition typically exerts a relapsing course, which could have accounted for the patient’s initial presentation and subsequent sudden rapid deterioration. Several other disorders were considered; however, investigations were unable to confirm any of the diagnoses suspected (Table 1).

**Table 1:** Diagnoses considered in this case.

Diagnosis considered	Investigations ordered
Neuromyelitis optica	Anti-NMO
Sjorgen’s syndrome	ANA, SSA/Ro, SSB/La
Systemic lupus erythematosus	ANA, SM, SSA/Ro, SSB/La, U1RNP
Mixed connective tissue disease	ANA, U1RNP
Antiphospholipid syndrome	Anticardiolipin
Systemic sclerosis	ANA, anticentromere, Scl-70
Neurosarcoidosis	CSF ACE
Paraneoplastic myelitis	Anti-Hu
Acute viral myelitis	PCR for HBV, HCV, HSV1/HSV2, enterovirus

*The differential diagnosis is wide and includes inflammatory, vascular, neoplastic, and infectious diseases*.

It is likely that the patient’s fall occurred as a consequence of the spinal cord subacute ischemic change and that the subsequent deterioration was due to the progression of the spinal ischemia to frank spinal cord necrosis with rostral extension to the cervicomedullary junction. A similar vasculitic process may have been the reason behind the patient’s initial presentation with suspected transverse myelitis. Despite extensive investigation, no systemic etiology was discovered. Indeed the autopsy did not reveal any involvement outside of the CNS. More so, the vasculitic features were confined to the spinal cord, sparing the brain. PACNS predominantly affects small and medium-sized arteries of the brain, spinal cord, and leptomeninges. It is rare, and when described does not usually involve the spinal cord, although it has been described (3). Isolated spinal cord vasculitis was described on very few occasions. Feasby et al. (4) described a fatal myelopathy with numerous areas of old and recent necrotic and vasculitic changes in branches of the anterior spinal artery and in the posterior spinal arteries. Ropper et al. (5) reported another fatal case with a predominantly lymphocytic leptomeningeal vascular infiltration most strikingly in branches of the anterior spinal artery. Goertz et al. (6) reported a case of a methotrexate responsive myelopathy where a spinal biopsy was obtained and demonstrated vascular thrombosis and transmural inflammation consisting of lymphocytes, epitheloid cells, and giant cells. In this case, there was a degree of steroid response initially, which supported the presumed diagnosis of demyelination. Rourke et al. (7) reported a case of progressive paraparesis where an MRI revealed a signal abnormality extending from the third thoracic level to the conus, while brain MRI was normal. Neoplastic disease was suspected and a biopsy was obtained revealing evidence of vasculitis in small and medium-sized vessels.

At the time when deterioration occurred and subsequently CSF analysis showed marked proteinorrhacia and pleocytosis, cell injury was probably irreversible. Immunosuppression was given through a process of elimination, and an accurate diagnosis would have made little difference to the outcome. However, when the patient first presented, imaging and laboratory studies were equivocal. A selective catheter angiography (8) may have revealed evidence of vasculitis. Spinal cord biopsy would have been needed to obtain an accurate diagnosis. These tests would have required long distance transfer and were only practical and potentially influential on treatment prior to the deterioration in the patient’s condition. Unfortunately, vasculitis was not considered at that stage.

## Ethics Statement

Written informed consent for the publication of this case report was obtained from the patients’ next of kin.

## Author Contributions

AF and MF wrote the manuscript. HR and MH cared for the patient. All the authors reviewed and approved the manuscript.

## Conflict of Interest Statement

The authors declare that the research was conducted in the absence of any commercial or financial relationships that could be construed as a potential conflict of interest.
